# Fixation Release and the Bone Bandaid: A New Bone Fixation Device Paradigm

**DOI:** 10.3390/bioengineering4010005

**Published:** 2017-01-22

**Authors:** Narges Shayesteh Moghaddam, Ahmadreza Jahadakbar, Amirhesam Amerinatanzi, Roman Skoracki, Michael Miller, David Dean, Mohammad Elahinia

**Affiliations:** 1 Dynamic and Smart Systems Laboratory, The University of Toledo, Toledo, OH 43606, USA; narges.shayestehmoghaddam@rockets.utoledo.edu (N.S.M.); ajahada@rockets.utoledo.edu (A.J.); amirhesam.amerinatanzi@rockets.utoledo.edu (A.A.); 2 Department of Plastic Surgery, The Ohio State University, Columbus, OH 43210, USA; Roman.Skoracki@osumc.edu (R.S.); Michael.Miller@osumc.edu (M.M.); David.Dean@osumc.edu (D.D.)

**Keywords:** mandible, reconstructive surgery, finite element analysis, Bone Bandaid

## Abstract

The current gold standard of care for mandibular segmental defeat reconstruction is the use of Ti-6Al-4V immobilization hardware and fibular double barrel graft. This method is often successful immediately at restoring mandible function, however the highly stiff fixation hardware causes stress shielding of the grafted bone and stress concentration in the fixation device over time which can lead to fixation device failure and revision surgery. The purpose of reconstructive surgery could be to create normal stress trajectories in the mandible following engraftment. We investigate the use of a two stage mechanism which separates the immobilization/healing and regenerative phases of mandibular segmental defect treatment. The device includes the use of a very stiff, Ti-6Al-4V, releasable mechanism which assures bone healing. Therefore it could be released once the reconstructed boney tissue and any of its ligamentous attachments have completely healed. Underneath the released Ti-6Al-4V plate would be a pre-loaded nitinol (NiTi) wire-frame apparatus that facilitates the normal stress-strain trajectory through the engrafted bone after the graft is healed in place and the Ti-6Al-4V fixation device has been released. Due to the use of NiTi wires forming a netting that connects vascularized bone and possibly bone chips, bone grafts are also more likely to be incorporate rather than to resorb. We first evaluated a healthy adult mandible during normal mastication to obtain the normal stress-strain distribution. Then, we developed the finite element (FE) model of the mandibular reconstruction (in the M1-3 region) with the proposed fixation device during the healing (locked state) and post-healing (released state) periods. To recreate normal stress trajectory in the reconstructed mandible, we applied the Response Surface Methodology (RMS) to optimize the Bone Bandaid geometry (i.e., wire diameters and location). The results demonstrate that the proposed mechanism immobilizes the grafted bone in the locked state properly since the maximum resultant gap (21.54 micron) between the graft and host mandible surfaces are in the safe region (less than 300 micron). By considering the von Mises criteria for failure, FE analysis together with experimental studies (i.e., compressive and tensile testing on the inferior and superior fixation devices, respectively) confirm that the proposed fixation devices do not fail, showing safety factor of at least 10.3. Based on the Response Surface Methodology (RSM) technique, the optimal parameter values for the wires are achieved (0.65 mm and 1 mm for the superior and inferior wires, respectively) and the required level of preload on each wire are calculated (369.8 N and 229 N for the inferior and superior wires, respectively). The FE results for stress distribution on the reconstructed mandible during the released state closely match that of a healthy mandible.

## 1. Introduction

The current standard of care mandibular segmental defect reconstruction uses permanently placed Surgical Grade 5 (Ti-6Al-4V) titanium plates and a fibular double barrel graft, or sometimes in the case of small defects, an iliac crest bone graft. The purpose of the graft is to restore the bony mandible, integrate that bone with the gums, and provide seating for titanium dental implant posts which, once fully integrated in the bone, will receive ceramic crowns. The fibular or iliac bone graft should also integrate well enough to support chewing following mandibular reconstruction. Although Ti-6Al-4V fixation plates can successfully provide mandibular immobilization during the healing period, they often cause problems during the post-healing period. When compared to mandibular cortical bone stiffness, Ti-6Al-4V has a much higher stiffness. This high stiffness may alter the mandibular loading pattern previously seen during chewing and possibly shield the grafted and/or host bone from carrying a normal load [1]. Thus, the bone is shielded in regions previously stressed (i.e., stress shielding) and higher levels of stress may be applied in areas that had previously seen less stress (i.e., stress concentration). The shielded bone may undergo a remodeling process that results in bone resorption which may lead to catastrophic failures [2,3]. Fixation failures due to either stress shielding or stress concentration necessitate revision surgeries. These additional procedures would be avoided if materials with more useful mechanical properties are used for post-surgical skeletal fixation and if those devices have both material properties and geometries that supported the recreation of normal stress-strain trajectories [4,5].

Shayesteh Moghaddam et al. [6] proposed the use of a less stiff material, NiTi (nickel titanium or NiTi), for fixation devices with the same geometry used in current Ti-6Al-4V skeletal fixation devices. NiTi has attracted much attention due to its low stiffness, biocompatibility, corrosion resistance, and appropriate mechanical behavior [7,8,9]. The engineered porosity of NiTi devices can be designed to have stiffness levels equal to the cortical bone of the mandible, which is significantly less stiff than solid NiTi or Ti-6Al-4V devices [10,11,12]. Additive Manufacturing (AM) techniques have enabled the fabrication of engineered porosity within metallic implants [11,13,14,15]. This type of device offers: (1) increased stress on the bone graft which may spur remodeling and incorporation while at the same time prevent resorption, thereby possibly allowing it to support dental implants [16,17]; (2) reduced stress concentration on fixation plates and screws would reduce the risk of mechanical failure in these components. The low stiffness of a porous NiTi device could however adversely affect the immobilization needed during the healing process. It is, therefore, necessary to design a skeletal fixation device that not only recreates normal stress distribution between the grafted bone and the host bone, but also during the post-surgical healing period, but also immobilizes the graft against the host bone during the healing period.

We suggest in this study that it is possible to both initially immobilize and later reestablish normal stress trajectories in the host mandible as well as the grafted bone with a dual-purpose device. We refer to this device as the Bone Bandaid. It includes stiff Ti-6Al-4V components that can be released. In the released state a stiffness-matched NiTi wire-frame provides the previously mentioned function of long term redirection of load into normal directions that facilitate remodeling and result success of the fixation procedure [18]. In other words, the Ti-6Al-4V fixation hardware functions during the healing period, and the released NiTi webbing functions during the post-healing period. The highly stiff fixation hardware is needed to bring mandibular micro-motion below 300 micron during the most powerful chewing while the grafted bone is healing with the surrounding host mandible [19,20,21]. If the highly stiff fixation hardware is not released once the bone is healed, it would continue to alter the normal stress-strain trajectory seen during chewing and shield the engrafted bone from stress which could lead to its resorption [22,23]. As another adverse effect, the post-operative power loss of 40%–50% does not improve among patients when the grafted bone fails to heal, remodel, and strengthen as expected due to stress shielding [24].

With our device, the Bone Bandaid, the releasable mechanism effectively removes the fixation hardware from loading after the bone is healed. Following release of the device, the NiTi webbing acts as a superstructure, a skin, to the underlying grafted cortical bone. NiTi is a promising choice due to its superelastic behavior as explained in Section 3.3. We hypothesize that the NiTi web will facilitate the transduction of stress to the grafted bone thereby bringing about a normal stress-strain trajectory and allowing the restoration of muscle power. As a result of the normal stress-strain trajectories, bone remodeling will change the morphology of the grafted bone so that it can provide long-term strength that will be needed for dental implants. The Bone Bandaid’s NiTi web also acts as a failsafe in the event of modest levels of trauma, preventing disintegration and disruption of the surgically reconstructed site. The use of the web would also facilitate the incorporation of bone chips (i.e., onlay grafts). These grafts often resorb if they are placed in areas that have little load and a poor blood supply. When the chips are used in combination with a vascularized graft, the microsurgically implanted vascular fibular graft includes a vascular predicle (i.e., a blood supply) [25].

Normative mandibular stress patterns can be determined via Finite Element Analysis (FEA) [26,27]. Therefore, a finite element model can be used to design and evaluate various immobilization strategies. Patient-specific fixation offers the promise of restoring the normal stress distribution. The FEA-based models serve as the planning tool to evaluate the long-term likelihood of success by assessing the alteration of the stress profile in the treated anatomy. To this end, the design can be optimized to offer the required geometric and material properties. In this study, we initially investigate the bone healing requirements and in the second stage we use a comparison with a healthy mandible under normal chewing conditions.

## 2. Materials and Methods

### 2.1. Finite Element (FE) Analysis

#### 2.1.1. Modeling

**Figure 1:**
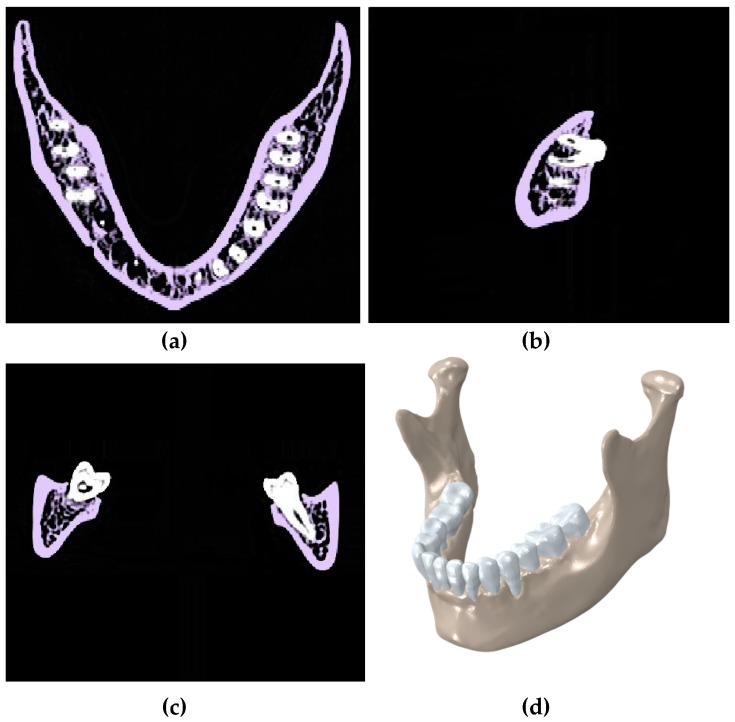
Segmentation of CT scan of a healthy mandible showing the (**a**) transverse plane; (**b**) sagittal plane; and (**c**) coronal plane; (**d**) a 3D model in Mimics.

A computer-aided model of a normal mandible (i.e., cadaveric bone for a healthy adult female, approximately 25–30 years of age) was created from CT scan data using the Mimics software 17.0 (Materialise, Plymouth, MI, USA). The healthy model included mandible cortices, cancellous, and teeth. Each component was segmented (i.e., extracted) and smoothed appropriately to remove CT slice-edges. Gap between the teeth and mandible of approximately 0.2 mm was assigned to a layer represent the periodontal ligament. These 3D reconstructed components were extracted as separated STL files from Mimics [28]. This model was later used as a reference model for comparing the stress-strain data (Figure 1).

**Figure 2:**
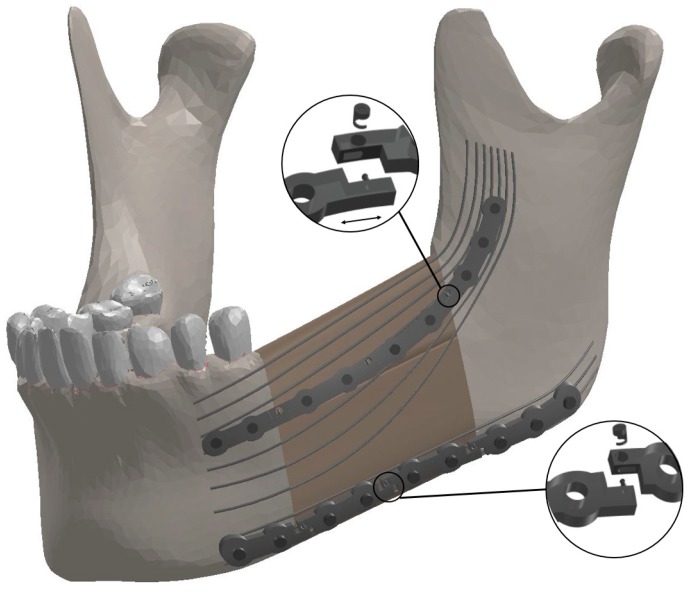
The components of the Bone Bandaid mandibular reconstruction using a releasable Ti-6Al-4V fixation device and underlying Bone Bandaid NiTi wires. The NiTi wires are affixed to the bone by tiny barbs that are not seen. Release is accomplished during a minimally invasive outpatient procedure using a sterile release microsurgical device. There are in total six release points. Since the upper fixation is under tensile loading, the mini-plates will move apart in released state, while the inferior ones get closer since they are under compressive loading.

To simulate the most common reconstructive surgery, a 40 mm mandibular segmental detect was resected on the left half of the mandible bearing M1-3. A fibular double barrel graft was virtually made out of two segments of one fibula autograft consisted from separate cancellous and cortical bone. The double barrel graft had a total length of 40mm, a width of 14 mm, and a height of 38 mm to fill the resected area. To affix the graft into the remaining mandible, two releasable fixation devices with the dimensions of 77 mm × 6.4 mm × 2 mm were designed in Solidworks (Dassault Systèmes, Waltham, MA, USA) by following the outside contours of the bone. It is notable that the general dimension of these fixation devices match those referenced in the literature [29]; each of these simulated fixation devices consists of 4 mini-plates which were connected together with releasable cam screws (Figure 2).

The release mechanism connects the mini-plates and has two components, a cam lock and a cam nut. The end of one mini-plate is equipped with a cam lock, and the end of the adjacent mini-plate has a slot for the cam lock and a hole through which the cam nut is inserted into the cam lock. For the superior fixation device, the cam nut was locked in place by twisting it in a counterclockwise direction. However, the cam nut pertains to the inferior fixation device was locked by twisting in a clockwise direction. The reason for this difference is that the superior fixation device undergoes extension deformation during chewing while, at the same time, the inferior one tolerates contraction deformation. Each of the two plates would be disengaged at the desired time (i.e., post healing) by twisting the cam nut in the opposite direction during a minimally invasive procedure. Once released, the cam lock can slide in the sleeve on the other plate freely. However, and the two plates would have limited movement, just enough so as not to draw load from the mandible. In this project’s simulation, the superior plates have separated from each other once they are released, while the inferior ones get closer after the cam nuts are unlocked. The cam nuts have a diameter of 0.5 mm.

Both of the fixation bars had nine threaded holes. Bicortical screws (i.e., long screws that fully insert into the buccal cortical bone and reach the lingual cortical bone) were considered to fix each device securely to the surrounding mandible and graft. These bicortical screws have a diameter of 2.5 mm. As part of the patient-specific design, the screws are carefully placed so as to not intersect the roots of the teeth. The NiTi wire web includes 3 inferior and 6 superior longitudinal NiTi wires each with a length of approximately 92 mm and a diameter of 3 mm. The NiTi wires have been purchased from Fort Wayne Metals Ltd. (Fort Wayne, IN, USA). This structure is designed in a way to be laid on the graft and surround the mandible. It is essential to optimize the diameter of each wire to result in a normal stress distribution along the reconstructed mandible. To this end, a Response Surface Methodology (RSM) was used (details in Section 2.3). The wires were hooked via small barbs into the graft and surrounding host bone at several points (Figure 2).

#### 2.1.2. Meshing

**Figure 3:**
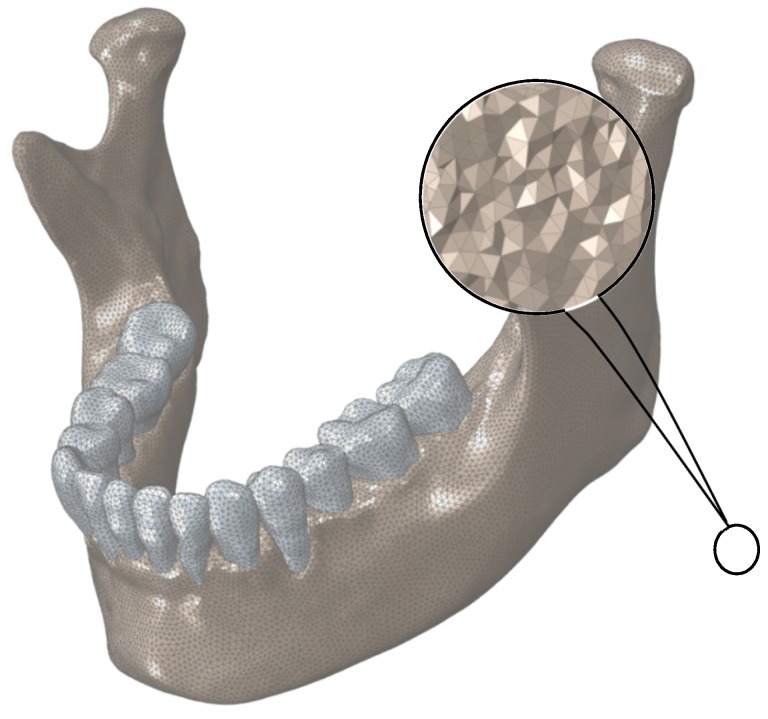
Finite Element mesh for normal mandible. The assigned mesh is 10-node tetrahedral elements (C3D10).

The 3D models of all components were meshed in Hypermesh (Hyperworks, Troy, MI, USA) with 10-node deformable, tetrahedral elements (C3D10) for more accurate results [30]. Figure 3 demonstrates the meshed healthy mandible.

**Figure 4:**
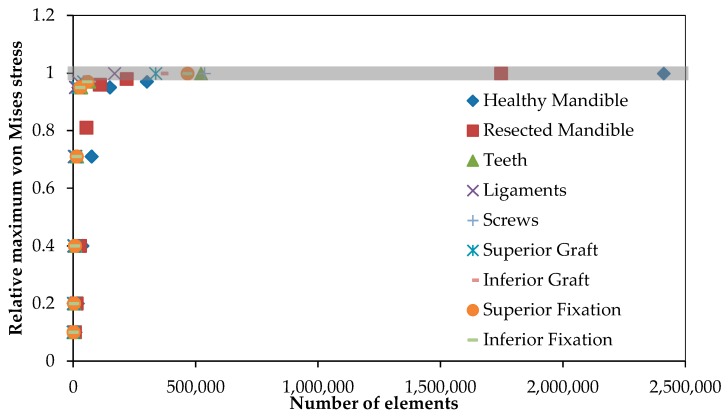
The mesh convergence study was done to define the optimized number of elements for each component. The vertical axis shows the maximum von Mises stress on each component relative to the maximum von Mises stress on the same component with the highest number of elements.

To refine the mesh size and numbers of elements, mesh convergence analysis of each component was conducted separately prior to assembling the model. In this analysis, seven different mesh densities were considered for each model component. Figure 4 represents the data that pertains to our convergence study. The optimum number of mesh elements for each component is also presented in Table 1.

#### 2.1.3. Material Properties

The meshed bone structure of mandible was imported into Mimics software to calculate the Hounsfield unit (HU) numbers (i.e., density) at each element in order to estimate the material properties. Using phantom calibration data, the HU numbers were converted into ash density (ρ_ash_) [31]. The derived ash densities ranged from 0.0085 g/cm^3^ to 0.0134 g/cm^3^. This range for density variation was subsequently divided into 10 equal subdivisions each pertains to one set of material properties. An isotropic modulus of elasticity was assumed for the bone structure. Using Equation (1) [31], the corresponding modulus of elasticity of each material set was achieved based on the derived ash density values:

(1) $$E=14,664\;\times \;\rho_{\mathrm{ash}}^{1.49}$$

**Figure 5:**
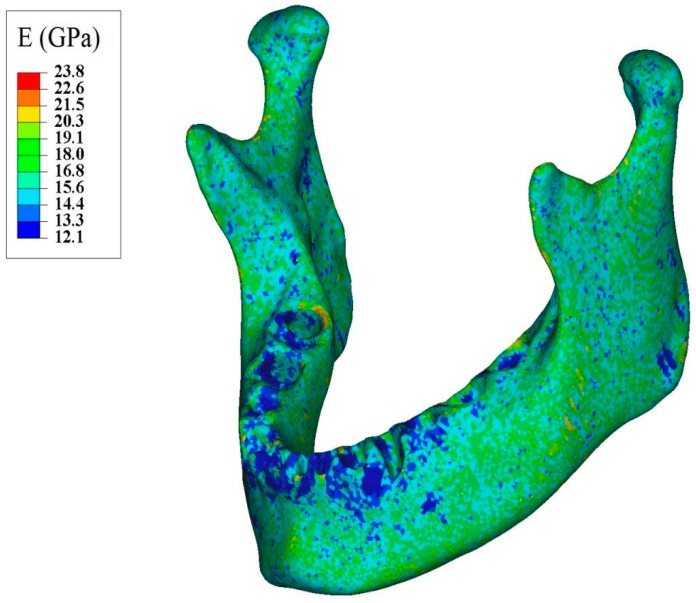
Apparent modulus of elasticity distribution throughout normal mandible assigned based on the ash density.

In addition, a constant isotropic Poisson’s ratio of 0.33 was assumed for the bone [31]. Finally, the corresponding densities and moduli of elasticity were assigned to each element. Figure 5 demonstrates the distribution of modulus of elasticity along the mandible. 

The material properties of fibula cortical and cancellous bone were obtained from an study by Moghaddam et al. [6]. The material properties of other components were taken from studies by Andani et al., Shetty et al., and Nagasao et al., which are summarized in Table 2 [32,33,34,35]. To assign the material properties of the NiTi wires, a validated user-defined material model (or UMAT) was used which required mechanical and thermomechanical properties of the superelastic NiTi. How the UMAT is calibrated and validated is fully explained in Section 3.1.1.

#### 2.1.4. Boundary Conditions

All of the meshed 3D structures with optimum mesh density were assembled together in Abaqus (CAE v6.11, Dassault Systems, Providence, RI, USA) and possible interactions between teeth-ligaments and ligaments-cortical bone were considered as tie [19,36,37]. The possible interaction between screw-fixation, screw-host mandible, screw-graft, teeth-ligaments and host mandible-ligaments were defined as tie constraints. Moreover, the friction factors of 0 and 1 are considered for the simulation of surface-to-surface contact between the host mandible and the fibular graft during healing and after healing, respectively.

To simulate the equivalent bite load, seven nodes on the buccal cusps of the lower right first molar were constrained. As a validation for such assumption, the summation of the reaction forces of these seven nodes were obtained while the muscle loads were gradually and proportionally increased. The resultant reaction force matched the bite force required to chew (i.e., a 526 N, error ~1%). In addition, 24 nodes on each tempromandibular joint was restrained from moving in all directions (the effect of articular disc is ignored) [21].

**Figure 6:**
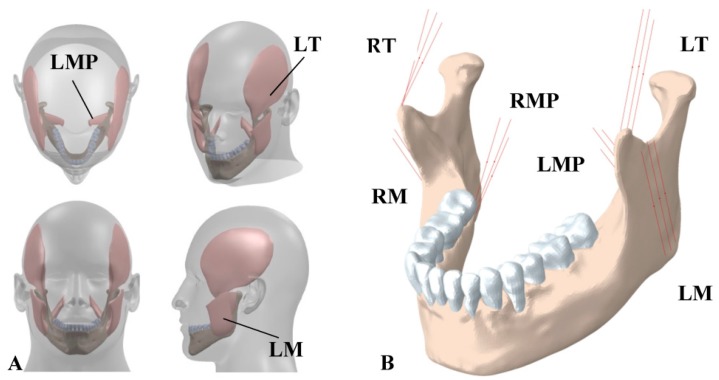
(**A**) 3D structures of major masticatory muscles; (**B**) Each muscle is simulated by three truss elements, considering the origin and insertion points taken from the 3D structures of muscles. RT = Right temporalis, LT = Left temporalis, RMP = Right medial pterygoid, LMP = Left medial pterygoid, RM = Right masseter, and LM = Left masseter.

The main chewing muscles are masseter, temporalis, and medial pterygoid. We adopted the baseline data for muscle forces provided by Korioth et al. [21]. Separate 3D model of each muscle was used to derive the initial direction of the associated muscle force, via indicating the coordinates of the origin and the insertion points (Figure 6A). Then, each muscle was simulated by three truss elements (T3D2) using the connector variable in Abaqus [38,39]. The origins of the truss elements were fixed while the insertion points were able to move during the chewing due to the jaw movement. Therefore, these assumptions allowed for the change in the direction of the muscle forces during chewing. In addition, since the muscles do not act in compression state, each of the truss elements was considered as incompressible element.

To effectively employ superelasticity, a preload was applied to each wire to ensure that the NiTi wires enter the plateau regions [40]. The preload level of each wire was calculated from multiplying the corresponding diameter by the required stress ((more details about the required stress can be found in Appendix A).

### 2.2. Optimization through Response Surface Methodology (RSM)

In order to recreate normal stress in the reconstructed and healed mandible (i.e., post-release), the NiTi structure was optimized using Response Surface Methodology (RSM). In this study, Central Composite Design (CCD) was used which is the most common RSM. This approach finds the optimized effective parameters and corresponding interaction between them by conducting minimum experiments [41]. Statistically, CCD is an experimental design for building a second order model for the RSM to obtain an optimal response.

The apparatus consists of nine wires, six superior and three inferior. To simplify our model, the diameter of the inferior wires was considered as the first effective parameter (X_1_), and that of superior ones was considered to be the second effective parameter (X_2_) influencing the resultant von Mises stress distribution in post healing period (i.e., released state). The length of each wire was assumed to be a constant (92 mm) based on the length of the resected area. The preload level of each wire was calculated from multiplying the corresponding diameter by the required stress (more details about the required stress can be found in Appendix A).

A major goal of this study was to determine the optimal parameters X_1_ and X_2_ that result in an average von Mises stress in different regions of the reconstructed mandible (i.e., alveolar, symphysis, body, angle, ramus, coronoid, and condyle) similar to those of a healthy mandible. The range of 0.6–3.0 mm was considered for each effective parameter, as seen in Table 3. The value for ∝ is dependent on the effective parameters in Equation (2):

(2) $$\alpha ={\left(N_{f}\right)}^{\frac{1}{4}}$$

where *N_f_* = 2K and K = the number of effective parameters.

By having the aforementioned range for each parameter, a total of nine different combinations of X_1_ and X_2_ were achieved. Then, the corresponding FE models were created and analyzed in Abaqus and the calculated values for average von Mises stress distribution in different regions of the reconstructed mandible (i.e., alveolar, symphysis, body, angle, ramus, coronoid, and condyle) were recorded. Then, the observed data were fitted into a second-order polynomial model and the regression coefficients were obtained in Minitab v 16 (Minitab Inc., State College, PA, USA). Finally, 3D plots and corresponding 2D contours of different regions were plotted, and subsequently the desired regions where the average von Mises stress is equal to that seen in a normal healthy mandible in the similar zone were defined and hachured on each 2D contour. Finally, the optimized diameters of inferior (X_1_) and superior (X_2_) wires were selected in a way that the resultant average von Mises stress in different regions of the reconstructed mandible is in the hachured zones.

### 2.3. Experimental Procedure

**Figure 7:**
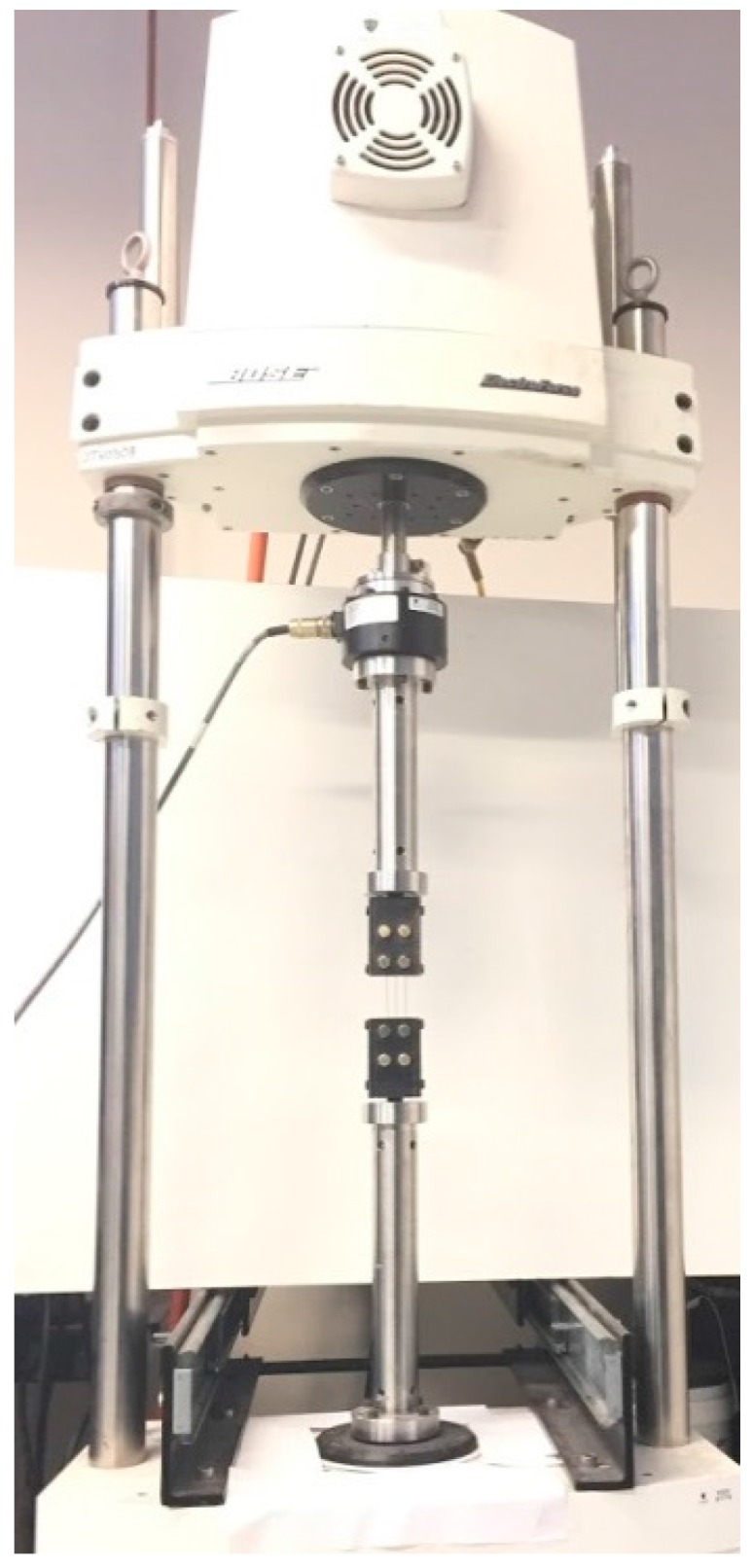
The experimental setup for tensile test on a Bone Bandaid NiTi wire.

Mechanical tests are performed on the Bone Bandaid apparatus at the strain rate of 0.006 s^−1^ at room temperature, using an electro-mechanical testing machine (Bose ElectroForce 3330). Figure 7 presents the experimental setup for the testing of Bone Bandaid apparatus.

## 3. Results

### 3.1. Validation

#### 3.1.1. User Defined Material Subroutine (UMAT)

**Figure 8:**
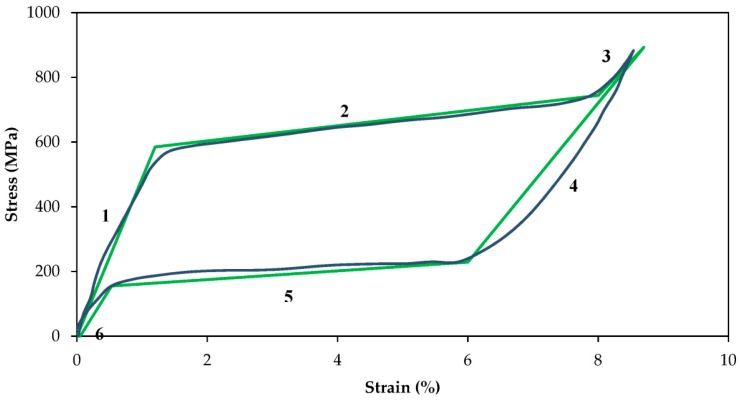
Stress-strain plots of superelastic NiTi wires and the validation of the UMAT.

The required inputs for calibration of the UMAT are Ms (martensitic start transformation temperature), Mf (martensitic finish transformation temperature), As (austenitic start transformation temperature), and Af (austenitic finish transformation temperature), CM (the slopes of martensite in the stress-temperature phase diagram), CA (the slopes of austenite in the stress-temperature phase diagram), EM (Young’s moduli in the martensite in the stress-strain curve), and EA (Young’s moduli in the austenite in the stress-strain curve). To obtain EA and EM, tensile tests were performed on a wire with 1mm diameter and 60 mm length at the strain rate of 0.006 s^−1^ at room temperature using a BOSE ElectroForce 3330 (New Castle, DE, USA) machine. Figure 8 depicts the stress strain plot obtained from the loading-unloading of a superelastic NiTi wire.

Using the plot, the slopes of the linear, fully transformed, regions of martensite (EM) and austenite (EA) material were calculated to be 30,000 and 40,000, respectively. The other required information, including Ms, Mf, As, Af, CM, and CA were taken from our previous studies and our readings of the literature, which were reported to be ࢤ65°C, ࢤ88°C, ࢤ23°C, ࢤ8°C, 5.7, and 8.6, respectively [42,43,44,45,46]. These material properties are summarized in Table 4. Using these material properties, the UMAT is calibrated and the tensile test is simulated. Figure 8 also demonstrates the results of the simulations using this UMAT. There is good agreement between the simulation and experimental results in the tensile test. This verifies the calibration of the UMAT and modeling of the NiTi wires.

#### 3.1.2. FE Model of the Mandible

**Figure 9:**
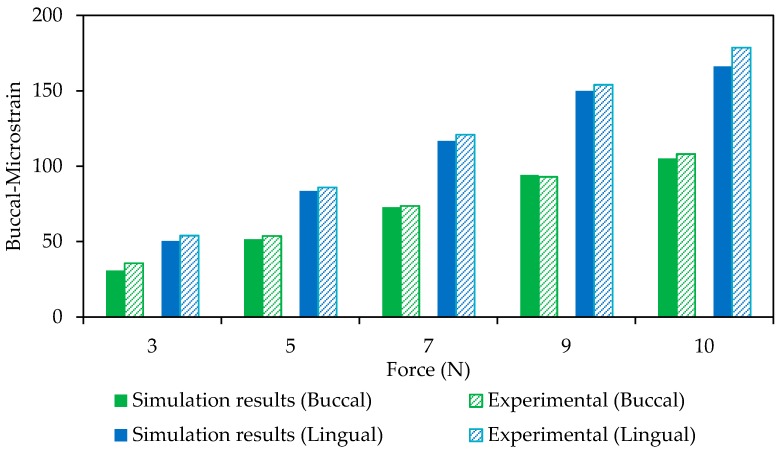
A comparison between FE model and experimental data on the buccal and lingual regions (Model Validation).

An experimental study was used as a reference for the validation of the finite element model of the mandible. The proposed model in this study was modified based on the reference experimental study and the results were compared to confirm the validation of the model. Ichim et al. [47] performed a mechanical test on a normal adult cadaver mandible and reported the strains on specific regions. In the reference study, the buccal and lingual strains under a normal loading were measured using different strain gauges. Subsequently, we have applied the same boundary and loading conditions to our model and reported the resultant $\frac{\Delta l}{l}$ for the similar regions to the reference study. The simulation and experimental results of the buccal and lingual regions of the mandible are shown in Figure 9.

As seen in Figure 9, the experimental and the finite element data are in an agreement with each other (Lingual side: *r* > 0.99, *p* < 0.0005, RMSE (root mean square error) < 6.42 × 10^−6^; Buccal side: *r* > 0.99, *p* < 0.0005, RMSE < 2.8 × 10^−6^).

### 3.2. Von Mises Stress Distribution on the Reconstructed vs. Healthy Mandible

The average von Mises stress in different regions of the reconstructed as well as normal healthy mandible (i.e., alveolar, symphysis, body, angle, ramus, coronoid, and condyle) were calculated and reported in Table 5. The results showed high discrepancy between the healthy and reconstructed model, which demanded the optimization of wire diameters. (Note: The diameters of the inferior and posterior wires in the reconstructed model were both considered to be 3 mm).

### 3.3. Optimization of the Design Parameters through RSM

#### 3.3.1. Central Composite Design (CCD) Analysis

The NiTi portion of the Bone Bandaid consists of nine wires, six covering the superior and the three covering the inferior areas of the graft bone and resected host mandible. All wires are pre-loaded prior to mounting on the reconstructed mandible (see more details in Appendix A). To simplify the optimization, the diameter of the six superior wires is considered as the first effective parameter, and that of the three inferior wires is considered as the second effective parameter.

Based on the RSM technique, a total of nine finite element analyses were conducted on the reconstructed model created with different combinations of superior and inferior wires and the resultant average von Mises stress in these two zones is reported in Minitab. Then, based on RSM analysis, the average von Mises stress in each zone was predicted as a function of the inferior ($X_{1}$) and superior ($X_{2}$) wires diameters and presented as following (Equation (3)):

(3) $$S_{\mathrm{avg}}=a+b\times X_{1}+c\times X_{2}+d\times X_{1}^{2}+e\times X_{2}^{2}+f\times X_{1}\times X_{2}$$

The coefficients were estimated by using linear regression and presented in Table 6.

The calculated average von Mises stresses in different zones are shown in Table 7. The predicted values of the RSM method are also provided in the table.

#### 3.3.2. Interactions between the Variables

**Figure 10:**
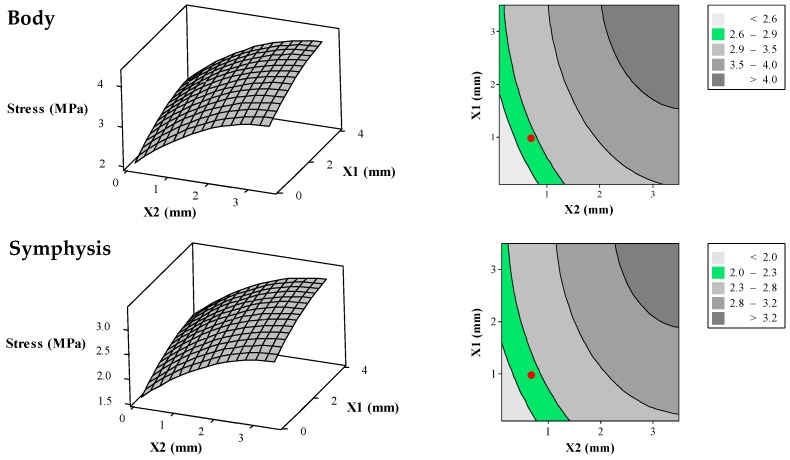

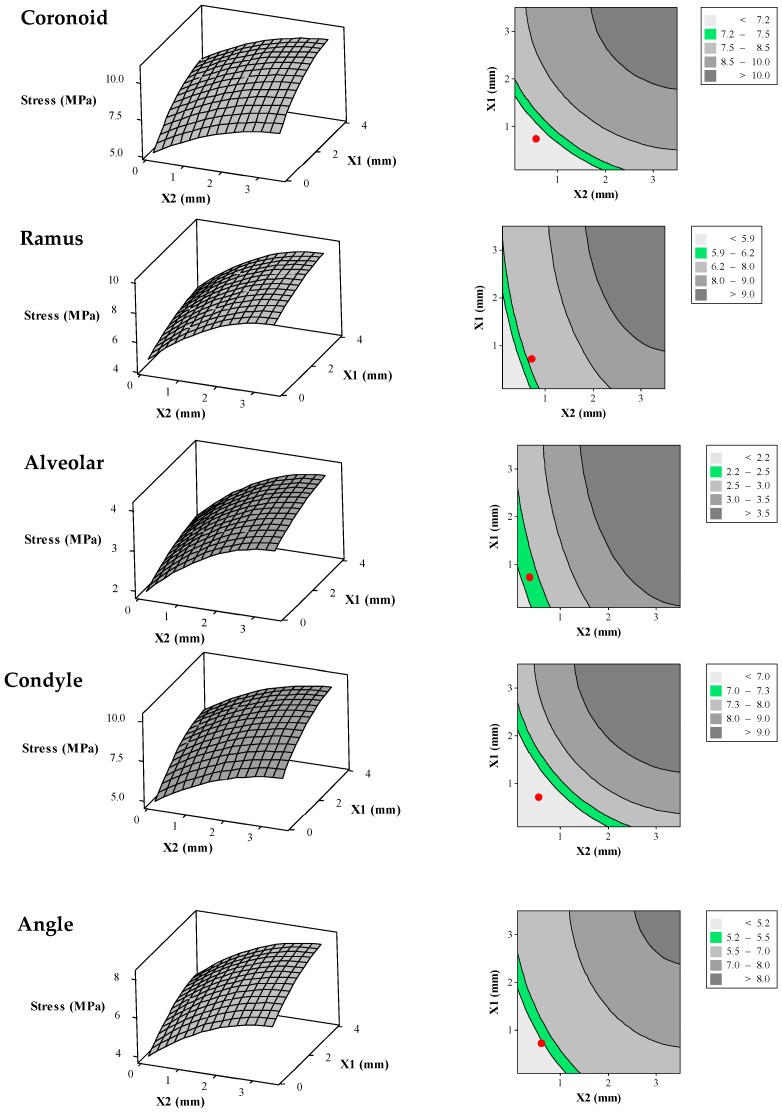
Surface plots (**left**) and their corresponding contour plots (**right**) representing average von Mises stress in the different zones of the reconstructed mandible (i.e., alveolar, symphysis, body, angle, ramus, coronoid, and condyle) as a function of the diameters of superior and inferior wires of the Bone Bandaid apparatus. In the 2D contour plots (**right**), the desired ranges for the average von Mises stresses are presented as green areas. The red circles are representative for the average von Mises stresses resulting from the combination of optimized X_1_ = 1 mm and X_2_ = 0.65 mm.

Figure 10 demonstrates the surface plots and their corresponding contour plots representing the average von Mises stress in different zones (i.e., alveolar, symphysis, body, angle, ramus, coronoid, and condyle) of the reconstructed mandible as the result of different diameters for superior and inferior wires. As it is clear Figure 10, the average von Mises stress increases by increasing wire diameter.

FE results for a normal, healthy mandible are also post-processed to calculate the average von Mises stress in similar zones which are as follows: Alveolar = 2.35 MPa, Symphysis = 2.15 MPa, Body = 2.75 MPa, Angle = 5.35 MPa, Ramus = 6.05MPa, Coronoid = 7.45 MPa, and Condyle = 7.15 MPa. In this study, an acceptable tolerance of 1.5 MPa is considered for each of these regions. That level is also used for the reconstructed mandible. These acceptable ranges are also hachured in 2D contours of Figure 10.

#### 3.3.3. Optimization Analysis

Based on the RSM results in Figure 10, the combination of 0.65 mm for the superior wires diameters and 1 mm for inferior wires diameters result in acceptable range for average von Mises stress for all regions except the Alveolar region (2.67 MPa compared to 2.41 MPa for healthy mandible) and condyle region (6.8 MPa compared to 7.13 MPa for healthy mandible). Since the possibility of bone resorption is low in these two regions, such differences are assumed to be negligible [4].

### 3.4. Evaluation of Reconstructed Mandible during Healing Period (Stage I: Locked State) Using Optimum Parameters

**Figure 11:**
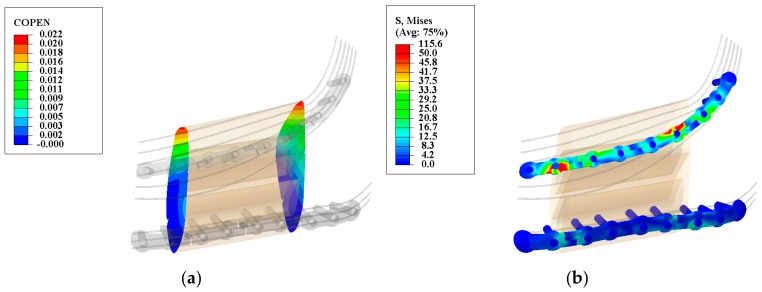
Deformation map for the graft/mandible junction (**a**) and von Mises stress distribution along the releasable Ti-6Al-4V hardware (**b**) in the second stage. The observed gap is less than the allowable gap (200–400 μm) and the maximum observed von Mises stresses are less than their yielding points (superior hardware: 1186.8 MPa, and inferior hardware: 1098.6 MPa). (Units: micron (μm) and megapascal (MPa)).

The success of healing period is measured by the safety of the fixation hardware (essential to avoid failure) while minimizing the gap between the host mandible and the grafted bone (necessary to ensure bone healing) [48,49]. At the interface between the grafted bone and host mandible the gap should be less than 300 micron [19,20] at all times, including the peak chewing load. The COPEN (i.e., contact opening) variable can be used in Abaqus to measure the relative position between the graft and host bone surfaces [50,51,52]. As seen in Figure 11a, the maximum gaps are 20.34 and 21.54 micron in the anterior and posterior regions of the graft bone, respectively. With these distances, it is expected that the healing progresses uninterrupted.

To check the safety, it is important to evaluate the maximum von Mises stress on the fixation device during the healing period. Figure 11b demonstrates that the maximum von Mises stresses on the superior and inferior fixation devices are 115.6 MPa and 50.0 MPa, respectively. 

**Figure 12:**
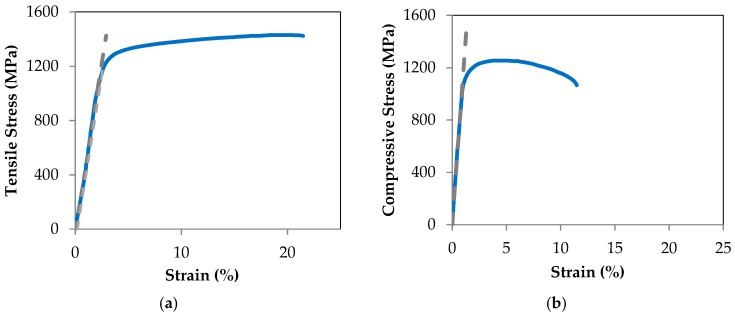
Tensile (**a**) and compressive (**b**) stress-strain plots for Ti-6Al-4V hardware. The tensile and compressive yield stresses by 0.2% offset strain technique are calculated to be 1186.8 MPa and 1098.6 MPa, respectively.

The superior fixation plate, often referred to as a “tension bar” undergoes tensile loading while the inferior plate, often referred to as the mandibular bar, is under high compressive loading. Figure 12a,b demonstrate the stress-strain plots for Ti-6Al-4V up to the failure during tensile and compression testing, respectively. The gray-dashed lines were used for calculating the Young’s modulus in tension and compression. In addition to the Young’s modulus, yield stresses for the tension and compression are calculated. Considering the von Mises Yield Criterion, the safety factors (SF) for yielding are 10.3 and 22.0 for the superior and inferior fixation devices, respectively.

### 3.5. Evaluation of Reconstructed Mandible in Post-Healing Period (Stage II: Released State) Using Optimum Parameters

**Figure 13:**
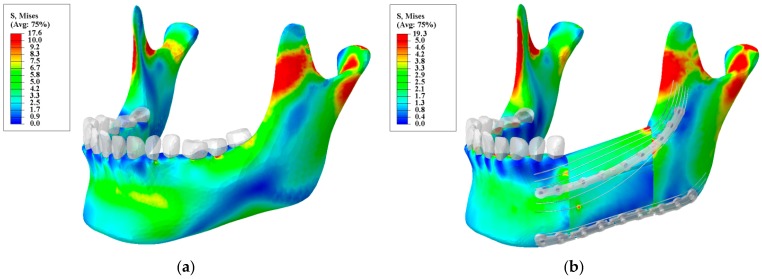
Von Mises stress distribution for: (**a**) a normal mandible; and (**b**) a reconstructed mandible with releasable Ti-6Al-4V hardware and underlying optimized NiTi Bone Bandaid wires. (Unit: megapascal (MPa)).

The Bone Bandaid’s post-healing period (i.e., released state) is intended to restore a normal stress distribution thereby a facilitatory as much restoration of muscle power and bone strength as possible. In this stage, the released device is no longer engaged and the NiTi wires are loaded along with the host and grafted bone. According to the optimization study, the optimum parameters for the NiTi wires are reported as following: Superior wires diameter = 0.65 mm, Inferior wires diameter = 1 mm, Wires length = 92 mm, Superior wires initial preload = 369.8 N, Inferior wires initial preload = 229N. It should be pointed out that this level of pre-loading was calculated in a way that the superior and inferior wires would be at the initiation point for their corresponding forward and reverse transformation, respectively (see more details in Section 3.3.2). Figure 13 depicts the stress distribution for stage II versus a normal, healthy mandible. It is shown that the stress-strain distribution is close to the normal mandible. It is, therefore, expected that the bone will remodel in response to this loading pattern. Then, would allow the reconstructed mandible to become stronger, thereby provide better support for dental implants. Although currently chewing power is typically reduced 40% in these patients, after the healing period the new Bone Bandaid device is expected to restore more of the normal loading capacity than is likely with current standard-of-care fixation following surgery.

**Figure 14:**
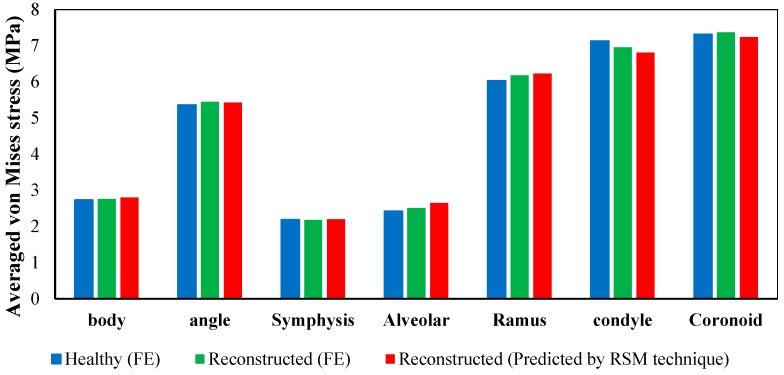
Average von Mises stress in different regions obtained from finite element analysis of a normal adult mandible, finite element analysis of reconstructed mandible, and predicated values for reconstructed mandible from the second order function estimated by RSM technique.

Figure 14 quantitatively compares the average von Mises stress in different zones of the normal mandible versus reconstructed one. The results show good agreement with each other (the maximum discrepancy of 4.6% on the condyle zone).

## 4. Discussion

The current standard of care for mandibular reconstruction is the use of permanent, non-porous Ti-6Al-4V fixation devices and a fibular double barrel graft. These standard-of-care fixation devices have a much higher stiffness than the surrounding cortical bone. Although this highly stiff fixation hardware allows bone to heal, it will continue to alter the normal stress-strain trajectories once the bone is healed. Previous studies have demonstrated the stress shielding effect, using FE analysis, on a reconstructed mandible with standard of the care Ti-6Al-4V fixation plates [4]. In a recent work, the use of stiffness matched porous fixation plates have been proposed for the reconstruction surgery to address the stress shielding problem [25]. However, using low stiffness plates decreased the level of immobilization during the healing period. To the best of authors knowledge, no work has been performed to design a fixation hardware that simultaneously provides immobilization, and restores normal chewing following bone healing.

In this study, we investigate the use of a two stage mechanism which de-links initial reconstructed (graft) bone healing from bone restoration and regeneration of masticatory power. The components of this new fixation and regenerative mechanism are (1) a releasable Ti-6Al-4V bone fixation device which is functional during the initial healing period and (2) an internal wire-frame apparatus, a NiTi webbing which is functional once the bone is healed. During the healing period, the highly stiff fixation hardware provides sufficient fixation for grafted bone to heal to the host bone. The breakthrough element of this new device is the ability to unlock this bone fixation in an outpatient procedure once the bone healing has occurred. The unlocking of the immobilization hardware allows the NiTi webbing to take over, to redirect the stress-strain trajectory in normal directions. The resumption of a normal stress-strain trajectory facilitate bone remodeling and strengthening of the graft, it should improve post-operative power in chewing, and it should improve the stability of dental implants placed in the grafted bone.

This study had several limitations that worth being addressed. The ideal goal for the optimization was to have a stress distribution close to that of a healthy mandible in different regions. Although a normal stress distribution was achieved in most regions of the reconstructed mandible, a discrepancy was observed on the condyle and alveolar region. It is desired to further optimize the model to meet the requirements for these two regions as well.

Another limitation in this study was the formula utilized to estimate Young’s modulus of elasticity according to the derived ash densities. There exists no formula reported specifically for the mandible in literature. Therefore, we employed a validated formula existing for femoral bone [53,54,55,56]. This should be investigated in the future studies.

## 5. Conclusions

In this work, we have proposed a two-stage process to address the competing needs of immobilization during healing period and re-establishment of normal stress-strain trajectories in grafted bone once the bone is healed. Our FEA for healing period (stage I: locked state) suggests that post reconstruction healing should occur, since the maximum gap between the engrafted bone and the remaining host mandible during chewing is 21.54 micron, less than the allowable 300 μm gap. According to compressive and tensile stress-strain plots for Ti-6Al-4V hardware, the superior and inferior releasable Ti-6Al-4V devices demonstrate safety factors of 10.3 and 22, respectively (Section 3.1.2). Using Response Surface Methodology (RSM), we optimized the second stage in a way to recreate stress distribution in every part of the reconstructed mandible, except alveolar and condyle regions, similar to that seen in a normal, healthy mandible. It should be pointed out that the observed discrepancy between the reconstructed and healthy mandible in these two regions were up to 4.6%. The optimized parameters for the Bone Bandaid NiTi wires were reported to be: Superior wires diameter = 0.65 mm, Inferior wires diameter = 1 mm, Wires length = 29 mm, preload on the superior wires = 369.8 N, preload on the inferior wires = 229 N. Using the optimum set of parameters, our FE results for post-healing period (stage II: released) demonstrate that stress-strain trajectories along the grafted bone and mandible are similar to those of a normal, healthy mandible. These results also confirm that normal chewing force would likely be restored for the reconstructed mandible. Finally, the experimental results on the Bone Bandaid apparatus confirm the performance of the model.

## Figures and Tables

**Table 1:** The number of elements for the Finite Element Analysis model components. The number of tetrahedral elements was determined by convergence analysis.

Component	Number of Elements
Healthy mandible	321,023
Resected mandible	218,328
Teeth (13 Total)	65,179
Ligaments (13 Total)	21,053
Top Graft	42,065
Lower Graft	45,037
Fixation hardware(s)	58,327
Screws (10 Total)	67,027

**Table 2:** Material properties of the Finite Element Model components. (Unit: megapascal or MPa).

Components	E (MPa)	υ
Cortical Fibular Graft	26,800	0.3
Cancellous Fibular Graft	1650	0.3
Ti-6Al-4V	112,000	0.3
NiTi	* 40,000	0.33
	** 30,000	
Teeth	17,600	0.25
Periodontal Ligament	2.7	0.45

* Austenitic modulus of elasticity; ** Martensitic modulus of elasticity.

**Table 3:** The ranges of effective parameters.

Variables, Unit	Factors	Range and Levels				
		−α	−1	0	1	Α
Inferior wires diameter (mm)	$X_{1}$	0.103	0.6	1.8	3	3.497
Superior wires diameter (mm)	$X_{2}$	0.103	0.6	1.8	3	3.497

**Table 4:** Material properties of the NiTi wires purchased from Fort Wayne Metals Ltd. (Fort Wayne, IN, USA).

Variables, Unit	Values
$E_{A}$ (MPa)	40,000
$E_{M}$ (MPa)	30,000
$M_{f}$ (°C)	−88
$M_{s}$ (°C)	−65
$A_{s}$ (°C)	−23
$A_{f}$ (°C)	−8
$C_{M}$ (MPa/°C)	5.7
$C_{A}$ (MPa/°C)	8.6
$\varepsilon_{L}$	0.039

**Table 5:** Average von Mises stress in different regions obtained from finite element analysis of the healthy mandible vs. the reconstructed mandible (MPa).

Mandible Type	Body	Angle	Symphysis	Alveolar	Ramus	Condyle	Coronoid
Healthy Mandible	2.37	5.36	2.184	2.42	6.03	7.13	7.31
Reconstructed	4.17	8.00	3.27	3.98	9.65	9.92	10.52

**Table 6:** The achieved coefficients from Response Surface Methodology (RSM) technique to predict the average von Mises stress $S_{\mathrm{avg}}$ in each zone.

Mandible Zones	a	b	c	d	e	f
Body	1.92	0.42	0.85	−0.05	−0.11	−0.01
Symphysis	1.50	0.33	0.67	−0.04	−0.09	−0.00
Coronoid	4.48	1.45	1.52	−0.19	−0.20	0.00
Ramus	4.44	0.77	2.18	−0.09	−0.30	−0.2
Alveolar	1.83	0.31	0.90	−0.03	−0.12	−0.00
Condyle	4.85	1.71	1.51	−0.23	−0.20	−0.01
Angle	3.69	0.95	1.49	−0.12	−0.20	−0.00

**Table 7:** Predicted and observed values obtained from RSM technique (X_1_ = Inferior wires diameter, X_2_ = Superior wires diameter, O = Observe, i.e., calculated with Finite Element Analysis (FEA), P = Predicted, i.e., calculated by RSM).

Model Number	Factors		Average von Mises Stress in Different Zones of the Mandible													
	X_1_	X_2_	Body		Angle		Symphysis		Alveolar		Ramus		Condyle		Coronoid	
	O.	P.	O.	P.	O.	P.	O.	P.	O.	P.	O.	P.	O.	P.	O.	P.
**1**	1.80	0.10	2.45	2.60	4.88	5.16	1.92	2.04	2.22	2.38	5.39	5.76	6.46	6.63	7.03	7.34
**2**	1.80	1.80	3.67	3.67	7.04	7.04	2.88	2.88	3.50	3.50	8.49	8.49	8.73	8.56	9.26	9.26
**3**	1.80	3.50	4.10	4.07	7.81	7.75	3.21	3.18	3.95	3.91	9.59	9.48	9.54	9.30	10.05	10.01
**4**	0.10	1.80	3.02	3.13	5.60	5.82	2.36	2.45	2.99	3.08	7.25	7.48	6.55	6.72	6.76	7.10
**5**	3.00	3.00	4.17	4.22	8.00	8.09	3.27	3.31	3.98	4.02	9.65	9.76	9.92	9.83	10.52	10.64
**6**	3.50	1.80	3.90	3.91	7.55	7.55	3.06	3.06	3.68	3.70	8.93	8.96	9.51	9.28	10.14	10.09
**7**	0.60	0.60	2.79	2.63	5.35	5.05	2.19	2.06	2.66	2.51	6.45	6.08	6.64	6.13	7.03	6.63
**8**	0.60	3.00	3.69	3.67	6.94	6.87	2.89	2.87	3.60	3.59	8.73	8.71	8.31	8.02	8.68	8.52
**9**	3.00	0.60	3.27	3.18	6.41	6.26	2.56	2.49	3.04	2.94	7.36	7.13	8.25	7.95	8.87	8.75
**R-Square**			97.53%		97.48%		97.53%		97.62%		97.64%		97.43%		97.46%	

**Figure A1:**
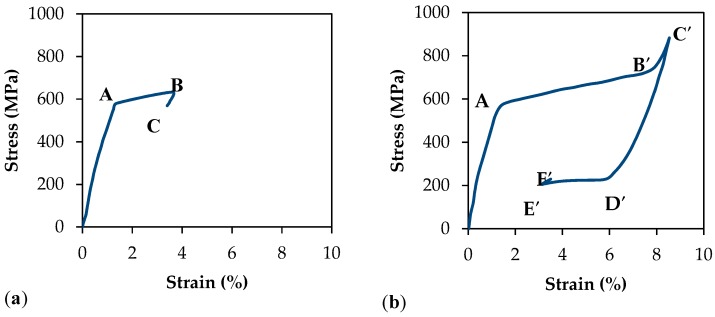
Stress-strain plots of superelastic NiTi wires: (**a**) The behavior of the superior wires during chewing (AB) and at rest (point C); (**b**) the behavior of the inferior wires during chewing (D’E’) and at rest (point F’).

## Appendix A

In general, during the normal chewing, the superior and inferior barrel grafts are under tension and compression loading. This results in an addition extension for the superior wires and a contraction for the inferior ones. For the superior NiTi wires the preload has been selected in a way that loads the wires to the critical start stress of loading (point A). Therefore, when bite force is applied to the mandible, the level of loading on these set of wires increases and the stress goes to the expected plateau region (region AB). Therefore, during chewing, an almost constant level of loading is applied to the mandible. When the mandible is at rest, the level of loading in the superior wires decreases and the level of stress in them also reduces (point C). This behavior is demonstrated in Figure A1a.

For the inferior set of wires, the level of preload is selected to be the critical start stress of unloading (point D’). When bite force is applied to the mandible, the level of stress reduces in these inferior wires, while an almost constant level of loading is transferred to the mandible by these wires (region D’E’). When the mandible is at rest, the level of stress increases in the inferior set of wires (point F’). This behavior is demonstrated in Figure A1b.
